# Improving Cytomegalovirus-Specific T Cell Reconstitution after Haploidentical Stem Cell Transplantation

**DOI:** 10.1155/2014/631951

**Published:** 2014-04-24

**Authors:** Xiao-Hua Luo, Ying-Jun Chang, Xiao-Jun Huang

**Affiliations:** ^1^Department of Hematology, The First Affiliated Hospital of Chongqing Medical University, 1 Youyi Road, Yuzhong District, Chongqing 400016, China; ^2^Peking University People's Hospital and Peking University Institute of Hematology, Beijing Key Laboratory of Hematopoietic Stem Cell Transplantation, No. 11 Xizhimen South Street, Beijing 100044, China

## Abstract

Cytomegalovirus (CMV) infection and delayed immune reconstitution (IR) remain serious obstacles for successful haploidentical stem cell transplantation (haplo-SCT). CMV-specific IR varied according to whether patients received manipulated/unmanipulated grafts or myeloablative/reduced intensity conditioning. CMV infection commonly occurs following impaired IR of T cell and its subsets. Here, we discuss the factors that influence IR based on currently available evidence. Adoptive transfer of donor T cells to improve CMV-specific IR is discussed. One should choose grafts from CMV-positive donors for transplant into CMV-positive recipients (D+/R+) because this will result in better IR than would grafts from CMV-negative donors (D−/R+). Stem cell source and donor age are other important factors. Posttransplant complications, including graft-versus-host disease and CMV infection, as well as their associated treatments, should also be considered. The effects of varying degrees of HLA disparity and conditioning regimens are more controversial. As many of these factors and strategies are considered in the setting of haplo-SCT, it is anticipated that haplo-SCT will continue to advance, further expanding our understanding of IR and CMV infection.

## 1. Introduction

Haploidentical stem cell transplantation (haplo-SCT) is an alternative treatment for transplant candidates lacking a human leukocyte antigen- (HLA-) matched related or appropriate unrelated donor. After hematopoietic stem cell transplantation (HSCT), T cells are regenerated through thymic and peripheral pathways, with the thymus generating a more diverse T cell repertoire. Because thymic function is poor in adults, posttransplantation immune reconstitution (IR) in the months following transplant depends on the peripheral expansion of mature T lymphocytes in the allograft. Impaired recovery of adaptive immunity following haplo-SCT remains an outstanding issue and is associated with increased risk of infection, including bacterial, fungal, and cytomegalovirus (CMV) infections. CMV infection after haplo-SCT continues to adversely affect transplant outcomes [1–4] despite the use of prophylactic or preemptive treatment [5]. Lack of CMV-specific immune recovery has been reported as consistently associated with relapses of CMV infection and the development of CMV disease after allogeneic stem cell transplantation [6–9]. Therefore, this review summarizes the kinetics of CMV-specific T cell recovery and its association with CMV infection after haplo-SCT. Strategies to improve CMV-specific IR are also discussed.

## 2. Cytomegalovirus-Specific T Cell Immune Reconstitution after Haplo-SCT (Table 1)

### 2.1. Manipulated (T Cell Depleted, TCD) Haplo-SCT

Using a megadose of extensively T cell depleted, G-CSF-mobilized stem cells and a fludarabine-based conditioning protocol [10], the Perugia group demonstrated that haplo-SCT could be successful in patients with acute leukemia. Early results [2] showed a nonrelapse mortality rate of 40%, with infection as the leading cause of death, mainly CMV and* Aspergillus*. Additionally, improved IR and fewer deaths secondary to infection occurred when G-CSF was eliminated from the regimen [11]. The results showed that in patients not treated with G-CSF, CD4+ cell counts were greater than 0.1 × 10^9^/L 60 days after transplantation and greater than 0.3 × 10^9^/L at 180 days. Subsequently, Lilleri et al. [12] performed a study with 48 young patients who received a TCD, allogeneic myeloablative HSCT from an HLA-disparate relative. The 1-year cumulative incidence of both CMV infection and specific T cell reconstitution was 83% among the 23 CMV-seropositive patients, and these incidences were 4% and 8%, respectively, among the 25 CMV-seronegative patients [12]. CMV-specific T cell (CMV-CTL) reconstitution was significantly delayed in patients receiving TCD grafts compared with patients receiving unmanipulated HSCTs [12].

Reduced intensity conditioning (RIC) is used to minimize toxicity while allowing rapid engraftment and expediting immune reconstitution during the early posttransplant period, thereby protecting the host from infection. Data showed that IR was rapid in 22 pediatric recipients after RIC and CD3-depleted haplo-SCT and was similar to, if not better than, outcomes obtained after myeloablative haploidentical transplantation [13]. CMV was detected in only one patient in this group, and no patient had died of viremia. In an attempt to reduce the risk of graft-versus-host disease (GVHD) and Epstein-Barr virus-related lymphoproliferative disease, Federmann et al. used CD3/CD19-depleted grafts with RIC and observed that T cell reconstitution after haplo-SCT was delayed with a median of 205 CD3+ cells/*μ*L, 70 CD3+ CD4+ cells/ul, and 66 CD3+ CD8+ cells/*μ*L on day 100, respectively [14]. Eight of the 28 patients had CMV reactivation, and no deaths due to infections were observed. Bader et al. reported their experience of CD3/CD19-depleted haplo-SCT for 22 children with acute leukemia [15]. Reconstitution with T cells can start on day 30 and the early T cell regeneration following transplantation results from the expansion of T precursor cells contained in the stem cell transplant. Thymus-dependent T cell regeneration only begins on day 100. In contrast to these published data, reports from Pérez-Martínez et al. using allogeneic CD3/CD19-depleted grafts showed that T cell recovery achieved normal values within the first 60 days after transplantation [16]. And up to 2 years, 2 of the 30 patients had died because of CMV pneumonia. Similar results were reported in patients with acquired severe aplastic anemia [17].

### 2.2. Unmanipulated Haplo-SCT

**Figure 1:**
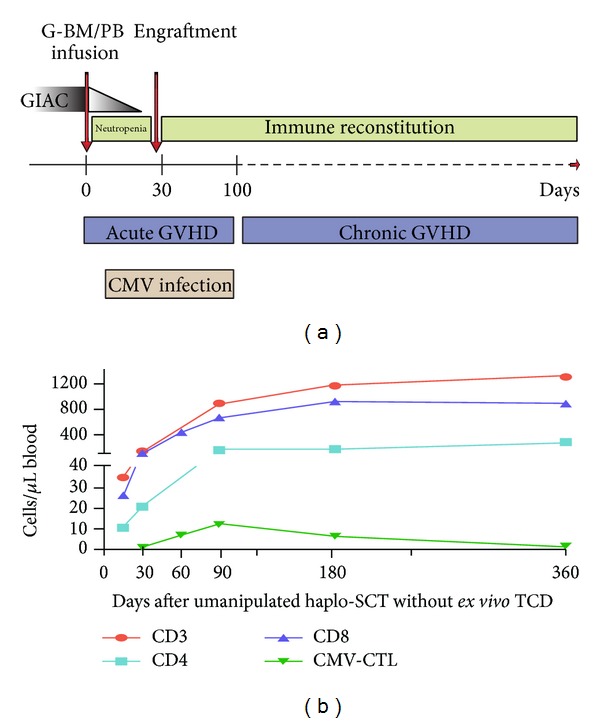
T cell immune reconstitution and CMV infection following unmanipulated haplo-SCT without* ex vivo* TCD (GIAC transplant protocol, Peking University Institute of Hematology). CMV, cytomegalovirus; GVHD, graft-versus-host disease; CMV-CTL, CMV-specific CTL; TCD, T cell depleted; G-BM/PB, combining G-CSF-primed bone marrow (G-BM) and peripheral blood (G-PB) harvests.

Although extensive depletion of T cells or selective depletion of alloreactive T cell subsets improves engraftment and reduces GVHD, this manipulation is associated with prolonged immune deficiencies and increased risk of infection. In an attempt to perform haplo-SCT without T cell depletion, Peking University researchers developed the GIAC protocol for haplo-SCT by combining G-CSF-primed bone marrow and unmanipulated PBSCs [18–22] (Figure 1). Using this protocol [23], we previously observed that patients undergoing haplo-SCT had a higher 100-day cumulative incidence of CMV antigenemia compared with a matched group (65% versus 39%), whereas the incidence of CMV-associated interstitial pneumonia was the same between the two groups (17% versus 17%). We investigated IR in patients with hematological malignancies after haploidentical transplantation and HLA-matched transplant [21]. Compared with those from HLA-matched recipients, T cell subset cell counts in the first 90 days after grafting were lower in haploidentical recipients. The difference was most striking for CD4+ and CD4+ naïve T cells. T cells appeared equally functional among patients without GVHD from both groups. Furthermore, we prospectively investigated CMV-CTL IR in 42 recipients after haplo-SCT [22]. CMV reactivation occurred in 36 of the 42 patients, but only 5 had CMV disease. The CD8+ T cell count in transplant recipients was equal to that of controls at day 60 after transplantation. The median number of CMV-specific T cells and the subsets to which they belonged was comparable to those of the controls from day 30 to day 360. Furthermore, CMV-CTLs from transplant recipients were found at high frequencies and demonstrated robust proliferation capacities and interferon-*γ* responses at 1 year after transplantation.

Recent reports showed that it is feasible to perform haplo-SCT without* ex vivo* TCD after RIC. Kurokawa et al. from Japan [24] conducted haplo-SCT on 66 adults with hematologic malignancies using RIC without TCD. CMV antigenemia occurred in 45 of 57 evaluable patients at a median of 19 days after transplantation. CMV-related diseases were diagnosed in 3 patients, and one patient died of CMV-colitis. The lowest numbers of CD3+, CD4+, and CD8+ T cells were observed at 1 month after transplantation, but all values continued to increase until 6 months after transplantation and remained stable thereafter [24]. Data from a Korean study [25] showed a RIC therapy with busulfan, fludarabine, and antithymocyte globulin (ATG) for haplo-SCT in acute leukemia and myelodysplastic syndrome. Fifty-eight of 72 evaluated patients (81%) had at least 1 positive assay result for CMV pp65 antigenemia. Four patients developed CMV disease, and 3 of them died of CMV-colitis per se or of other causes. Despite the use of ATG, CD8+ lymphocyte counts exceeded pretransplantation levels at 2 months, whereas CD4+ lymphocyte counts recovered more slowly, with only approximately half of all patients showing CD4+ lymphocyte counts > 200/*μ*L at 2 to 6 months after transplantation [25].

Alemtuzumab, which has a strong lympholytic effect, is usually used against GVHD in a reduced intensity conditioning regimen. Using an* in vivo* alemtuzumab-based regimen, Kanda et al. [26] reported that CD3+/CD4+ and CD3+/CD8+ T cells were strongly suppressed within 2 months after haploidentical peripheral blood SCT but recovered on day 90. CMV-specific cytotoxic T lymphocytes were detected on day 90 after transplantation in two patients and represented 0.03% and 0.25% of CD8+ T cells, respectively, for each patient. Ten of the 12 patients experienced CMV reactivation, and CMV disease was observed in three patients but was not fatal. Rizzieri et al. [27] extended the prior work and reported the large series assessing outcomes and immune reconstitution in nonmyeloablative haplo-SCT for 49 patients with alemtuzumab-based regimen. Twenty-five percent of the patients experienced a severe infection, whereas 86% experienced reactivated CMV with CMV disease in seven patients. Quantitative lymphocyte recovery through expansion of transplanted T cells was noted by 3 to 6 months [27]. Recently, Kanda et al. [28] updated their transplant data with continued use of* in vivo* T cell depletion with alemtuzumab. Nine patients experienced positive CMV antigenemia with CMV disease in three patients, none of which was fatal. The numbers of CD4+ and CD8+ T-cells remained low within one year after HSCT. The median quantities of CMV-specific CD8+ T lymphocytes as measured by the tetramer-based assay were 0.05%, 0.01%, and 1.83% at 90, 180, and 365 days after HSCT, respectively.

## 3. Cytomegalovirus Infection Associated with T Cell Immune Reconstitution

IR of the immune subsets is likely to have the greatest impact on clinical outcomes after haplo-SCT [29]. In healthy CMV-seropositive individuals, high frequencies of CMV-specific CD4+ and CD8+ T cells that mediate control of viral reactivation can be detected [30, 31]. Both the quantity and quality of CMV-specific T cell recovery are essential for immune control of CMV infection following HSCT. A strategy of deferred antiviral therapy based on the presence of a detectable functional CMV-specific T cell response at the time of documentation of CMV DNAemia was clinically administered and allowed for the sparing of antiviral treatment in transplant patients [32, 33]. A recent phase II study by Blyth et al. showed that donor-derived CMV-specific T cells reduce the requirement for CMV-directed pharmacotherapy without increased GVHD after allo-HSCT [34].

In immunocompromised HSCT recipients, few patients with levels of CMV-specific CD8+ lymphocytes > 2 × 10^6^–10 × 10^6^/L developed CMV disease [35–37]. Both CD4+ and CD8+ CMV-specific IR are required for protection from recurrent activation [38–40], and an absolute CD4+ and CD8+ T cell response at day 60 may confer protection against viremia in young patients [41]. Borchers et al. [42] reported that the presence of CMV-CTL before day 50 and their expansion after reactivation appear to protect against recurrent CMV reactivation. In patients with uncontrolled reactivation, differentiated CMV-specific T cells of the late differentiation phenotype CD45RA+CD27−CD28− did not develop [37]. Furthermore, Lilleri et al. [12] found that detection of CMV-specific T cells also correlated with control of CMV infection after T cell depleted haplo-SCT.

In our own analysis [43], high CMV-CTL with terminally differentiated effector CD45RO−CD62L− (T_EMRA_) phenotype in the allografts was associated with reduced risk of CMV reactivation when sufficient CD45RO+CD62L− cells (T_EM_) were provided by infusion (≥0.208 × 10^6^/kg). Early after transplantation, there was significant expansion of CMV-CTL with central memory CD45RO+CD62L+ (T_CM_) phenotype when CMV was reactivated [23, 43]. We further investigated CMV-CTL in bone marrow (BM) after haplo-SCT. BM-resident CMV-CTLs displayed distinct phenotypes when CMV was reactivated [23], as there are more T_EMRA_ in the BM at day 360 after SCT and relatively higher T_Naive_ cells (CD45RO−CD62L+) in the BM at day 90 in patients with infection compared with those without infection. This result suggested that CTL in BM may play an important role in controlling CMV infection, as mature T cells in the BM play an essential role in maintaining normal peripheral T cell numbers, and CMV-CTL could therefore be more efficiently derived from the BM than from the PB [44, 45].

## 4. Factors Influencing CMV-Specific IR

The process of IR is influenced by patient- and transplant-related factors, such as donor and patient ages, primary disease, transplant type, conditioning regimen, stem cell source, HLA disparity, GVHD, and infection [46]. Not surprisingly, the intensity of immunosuppression and the degree of T cell depletion in transplant protocols, such as ATG or alemtuzumab, both critically affect the risk of CMV reactivation [47]. As for CMV-specific IR after haplo-SCT, there are several influences, except graft manipulation described above.

### 4.1. Donor and Recipient CMV Serostatus

CMV-negative recipients of grafts from CMV negative donors (D−/R−) rarely develop CMV infection and D− should be chosen when possible. Ljungman et al. reported that only acute GVHD grade II–IV and D−/R+ were significant risk factors for CMV disease after multivariate analysis [48]. D+/R+ transplants, on average, generate higher levels of multifunctional CMV-specific T cells and require less antiviral therapy compared with D−/R+ transplants [49]. D+/R+ patients had a lower cumulative incidence of CMV reactivation, recurrent reactivation, CMV disease, and mortality compared with D−/R+ patients [50]. Pretransplant human CMV infection of the recipient is a major factor driving human CMV-specific immune reconstitution [12]. Our previous data also suggested protective immunity could be transferred by infusion of CMV-CTL within allografts [43]. Nevertheless, Pietersma et al. found that reactivation of CMV infection occurred more frequently in patients receiving a CMV-positive graft but was less severe than in patients receiving a CMV-negative graft [51]. These data suggest roles for both virus and CMV-specific immunity present in the graft. Based on current knowledge, the use of D+/R+ transplant is preferred for improved IR, and D−/R− is preferred for decreased risk of CMV infection. Other donor/recipient combinations remain to be confirmed in clinical trials. Determining CMV serostatus and levels of CMV-CTL in the donor grafts may help in controlling CMV reactivation, which is closely correlated with immune reconstitution and differentiation of CMV-CTL subsets.

### 4.2. Stem Cell Source and Graft Composition

Numerous studies have compared IR during SCT using different stem cell sources. IR after peripheral blood stem cell transplantation (PBSCT) is generally characterized by faster myeloid and lymphoid recovery versus BMT [52–54]. Along with accelerated and sustained naïve CD4+ recovery, improved* in vitro* proliferative responses have been measured following PBSCT [52–54]. Hakki et al. suggested that BM as the source of stem cells resulted in delayed recovery of functional T cell immunity at 3 months after transplantation [39]. In the setting of HLA-matched sibling transplantation, recipients receiving PBSCT had lower risks of documented bacterial, fungal, and viral infection, including CMV viremia [52]. These data clearly indicate rapid T cell reconstitution and a lower incidence of CMV infection when PBSCT is used.

Transplantation using PBSCs with* ex vivo* TCD is the most common HLA-mismatched/haploidentical transplant approach in Europe and the United States [55]. In China, Peking University researchers combined G-BM and G-PB harvests (G-BM/PB) without* ex vivo* TCD for the GIAC protocol and achieved encouraging results [18–21]. Recently, unmanipulated PBSCT [56] and unmanipulated G-BM [57] have been accomplished in haplo-SCT settings with very encouraging results. However, limited data are available concerning CMV-specific IR after haplo-SCT. Lilleri et al. reported that children receiving T cell depleted transplants exhibited significantly delayed CMV-specific T cell reconstitution, and only D− and BM as a stem cell source were found to significantly delay CMV-specific T cell reconstitution [12]. A small comparative series showed better survival among patients who received T cell-replete transplants, with less viral infections, including CMV reactivation, and better immune reconstitution of T cell subsets compared with T cell-depleted haplo-SCT [58]. Reconstitution of CMV-specific T cell immunity may have proceeded at a faster rate in patients treated with our GIAC protocol than in patients described in other reports of haplo-SCT [23]. A differential pattern of T cell reconstitution is expected after* in vivo* TCD and* ex vivo* TCD haplo-SCT. In TCD haplo-SCT, the time lapse during IR, even in the absence of GVHD, is most likely lengthened by extensive* ex vivo* T cell depletion itself, while greater numbers of donor T cells cotransfused with allografts are not immediately eradicated by* in vivo* TCD. The effect of* in vivo* T cell depletion could be balanced by graft infusion at the time of transplantation [43, 59]. Therefore, using PBSCT or G-BM/PB is preferred for IR to CMV.

### 4.3. Conditioning Regimens

Although limited, studies comparing IR following myeloablative and nonmyeloablative regimens have been insightful. Maris et al. compared IR for one year after transplantation in 51 patients receiving HLA-matched PBSCT following nonmyeloablative conditioning with a reference group of 67 myeloablative recipients [60]. Both regimens demonstrated similar levels of total and subset-specific lymphocyte recovery, lymphoproliferative responses to viral stimulants, and in total and pathogen-specific antibody levels. Overall infection rates were significantly lower in nonmyeloablated patients, who also had lower rates of CMV infection coinciding with greater numbers of CMV-specific T cells at days 30 and 90. Data from Nakamae and colleagues showed [61] that residual host cells after nonmyeloablative SCT reduce progression to higher CMV viral load in transplant recipients; however, this effect does not appear to protect against serious complications of CMV. Recent results [62] showed that CMV reactivation was less common in the RIC group during the midrecovery period, while there was no difference during the late-recovery period. CMV disease is as much of a problem following nonmyeloablative transplantation as it is following myeloablative transplantation [61, 63].

### 4.4. GVHD and Steroid Administration

The deleterious effects of acute GVHD on T cell function are well established. GVHD inhibits T cell recovery through T cell apoptosis via activation-induced cell death, immunosuppressive cytokine production by regulatory cell populations, and direct damage to thymic epithelium and stroma [64, 65]. GVHD appears to adversely affect all levels of T cell function, from delaying T cell ontogeny and limiting TCR diversity to impairing cytokine production in recovered T cells. Multivariable analysis showed that patients receiving methylprednisolone had a 1.5 times higher risk of infection, with acute GVHD being another independent risk factor for infections after transplantation [66]. Steroids can suppress immune function by promoting the development of high IL-10-producing regulatory T cells and inhibiting GATA-3 phosphorylation [67, 68]. High-dose steroid use (≥2 mg/kg/d) predicts delayed recovery of functional T cell immunity at 3 months after transplantation [39]. Özdemir et al. [69] showed that steroid administration resulted in a significant impairment in CD8+ tumor necrosis factor *α* (TNF*α*) production but not a decrease in the frequency of CMV-specific CD8+ T cells. Corticosteroid treatment may favor active viral replication even in patients with CMV-specific T cells [12]. These findings have implications regarding the tapering of steroids in patients with active infections normally controlled by T cell responses, such as CMV disease.

### 4.5. Subclinical CMV Reactivation

It is known that CMV infection drives T cells to an effector phenotype in healthy individuals [70]. Subclinical CMV reactivation while on ganciclovir appears to be a potent stimulator of T-cell function [39]. Among patients who received ganciclovir at engraftment, those who had breakthrough antigenemia had significantly better recovery of T cell function at 3 months compared with patients who remained antigenemia negative [39]. In the setting of HSCT and the absence of high-dose steroids, low-level, short-term antigenemia may, in fact, have a protective effect by enhancing late immune function. CMV infection is required for the generation and/or maintenance of the CMV-specific T cell pool, and reactivation of latent virus was identified as the main factor leading to immune reconstitution [12, 41]. Our data also showed that CMV-CTLs with a central memory CD45RO+CD62L+ phenotype significantly expanded when CMV was reactivated [23, 43]. However, prolonged CMV reactivation may lead to exhaustion of T cells, as has been suggested for other antigens [71]. These studies suggested that subclinical CMV reactivation, but not persistent CMV reactivation, may be required for the reconstitution of CMV-specific T cell responses.

### 4.6. Age and Degree of HLA Disparity

Children may have a better capacity than adults to develop anti-CMV primary immune responses after HSC transplantation [41]. Patients <8 years of age demonstrate improved IR, with a probability ratio of 4.57, and this likely results in better reconstitution of CMV-specific CD4+ and CD8+ T cells [12]. Increased thymic function might be responsible for better immune reconstitution in younger children [72], especially when compared with adult patients in whom naive thymic emigrants have been reported to appear in the circulation only 6 months after receipt of a T cell depleted HSCT [73]. Recently, Azevedo et al. [74] investigated long-term IR after RIC based haplo-SCT with TCD, which followed by preemptive donor lymphocyte infusions (DLI). They found the proportion of naive and memory subsets in the recipients, both within CD8+ and CD4+ T cells, more closely resembled that observed in age-matched control subjects than in the donors. Their data [74] suggested that long-term IR was successfully achieved after haploidentical HSCT, a process that appears to have largely relied on de novo T cell production. IR after haplo-SCT is usually slower than that after matched-sibling donor or matched-unrelated donor transplants [75]; however, the impact of HLA disparity on CMV-specific IR after haplo-SCT remains uncertain.

## 5. Adoptive Immunotherapy to Accelerate CMV-Specific T Cell Immune Reconstitution

Any further reduction in CMV infection after haplo-SCT will only be achieved by hastening posttransplant IR. To improve posttransplant IR, various strategies of adoptive donor T cell immunotherapy have been investigated over the past years. However, T cell-based adoptive therapy is problematic in the adult haploidentical transplant setting, for alloreactivity still exists. Research is focusing on strategies to hasten IR by adding back broad-repertoire or pathogen-specific mature donor T lymphocytes after* ex vivo* depletion of antidonor alloreactivity [76, 77].

Amrolia et al. demonstrated an accelerated immune reconstitution in 16 patients who received adoptively transferred T cells that were allodepleted* in vitro* [78]. After 2 to 4 months, CMV-specific T cells and a broad V*β* T cell receptor repertoire could be observed, while the incidence of GVHD was low. Posttransplantation CD8-depleted DLI can also contribute to improved T cell recovery after haplo-SCT for the treatment of advanced hematologic malignancies, while reducing the incidence and severity of acute GVHD [79]. Despite the high incidence of CMV reactivation (82%), no patients developed CMV disease. Circulating CD3+/CD4+ T cells significantly increased at day 120 after DLI, while the expansion of CD3+/CD8+ was at a median value of 23/*μ*L. Preliminary studies using gene engineering of donor lymphocytes to deplete alloreactive T cells appear to be promising [80, 81], but larger-scale investigations are warranted to confirm the results.

Given high degree of mismatching makes cell immunotherapy impossible, Perruccio et al. [76] improved the immune recovery after myeloablative haploidentical SCT by the infusion of nonalloreactive clones specific for CMV and* Aspergillus*. Within 3 weeks of the immunotherapy infusion, CMV-specific CD4+ T cell clones were 404 ± 124 per 10^6^ cells, and IFN-*γ*-producing CMV-specific CD8+ cells were detected in normal frequencies. Of the 25 patients who received CMV-specific adoptive therapy, CMV reactivation was observed in only 7 patients, while thirty of the 33 control patients experienced repeated CMV reactivation. More recently, Perruccio et al. [82, 83] tested a photodynamic approach to purge DLI of alloreactive, but not pathogen-specific, donor T cells. Pathogen-specific responses to CMV were retained, although with a 19 ± 9.7 time reduction in frequency [83]. Not only did the researchers achieve the success of described prophylactic infusion, but Feuchtinger et al. [84] also treated 18 patients with refractory CMV infection after allo-SCT using polyclonal CMV-specific T cells. In 83% of cases, CMV infection was cleared or viral burden was significantly reduced. Viral control was associated with the* in vivo* expansion of CMV-specific T lymphocytes in 12 of 16 evaluable cases, without GVHD induction or acute side effects.

These manipulated DLI approaches are effective but expensive and labor intensive, and the effect on global IR is unclear. For a long time following transplant, allogeneic DLI can accelerate IR, treat infections, and provide a graft-versus-malignancy effect [85, 86]. Currently, we focus mainly on the infusion of G-CSF-mobilized peripheral blood progenitor cells. Previous studies have shown the multiple biological effects of G-CSF on peripheral blood stem cells, such as the ability to polarize T cells from Th1 to Th2 and the promotion of regulatory T cell and tolerogenic dendritic cell differentiation [87, 88]. Huang et al. [89] reported that G-CSF-mobilized peripheral blood progenitor cell infusion produces superior disease-free survival in patients who received unprimed lymphocytes for relapse after allo-HSCT, although the difference in the incidence of severe GVHD was not significant. We extended the use of DLI for the treatment of infections. Our preliminary data showed that DLI is an effective and safe therapy for EBV-associated PTLD after mismatched/haploidentical SCT [90]. Investigation of DLI for CMV infection and other opportunistic infections is underway. Until pathogen-specific T cells and/or alloreactive-depleted T cells are more readily available, unmanipulated, nonspecific DLI will continue to play a role in the treatment of uncontrolled infections and improvement of IR following haplo-SCT.

## 6. Conclusions

The current options for haplo-SCT present intrinsic challenges. In T cell depleted haplo-SCT, the minimal residual T lymphocytes in the grafts successfully prevent lethal GVHD without any posttransplantation immunosuppression, but the small number of T cells infused leads to delayed IR. In unmanipulated haplo-SCT, although the high T cell content of the graft potentially enhances the graft-versus-leukemia effect, recipients of unmanipulated grafts from alternative donors remain at risk of TRM for months/years after transplantation because of GVHD and its immunosuppressive treatments that antagonize T cell expansion and function. Delayed IR and increased risk of CMV infection remain critical problems early after transplantation, although long-term IR can successfully be achieved after haplo-SCT [74, 91].

**Figure 2:**
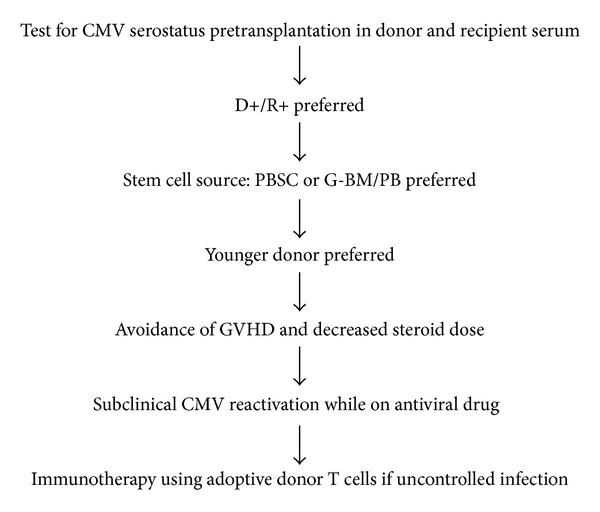
Proposed algorithm for improving CMV-specific IR following haplo-SCT. CMV, cytomegalovirus; D+/R+, CMV-positive recipients of grafts from CMV-positive donors; PBSC, peripheral blood stem cell; G-BM/PB, combining G-CSF-primed bone marrow (G-BM) and peripheral blood (G-PB) harvests; GVHD, graft-versus-host disease.

To address these shortcomings, several factors identified to affect IR to CMV should be considered for better outcome (Figure 2). Our data indicate that selection of D+ for R+, young donor, stem cells derived from PBSC or G-BM/PB, subclinical CMV reactivation while on antiviral therapy, avoidance of GVHD, and decreased steroid dose can improve CMV-specific IR. The effects of varying degrees of HLA disparity and conditioning regimens are uncertain. Therefore, more in-depth preclinical and clinical studies in this area are needed, both in terms of reconstitution of normal immune cell function and their effectiveness in anti-CMV cell activity.

## Figures and Tables

**Table 1:** CMV-specific immune recovery after haploidentical stem cell transplantation.

Group/Reference	Number	Disease	Graft manipulation	Conditioning	NRM or TRM	CMV infection	Immune reconstitution (IR)	Comments
TCD haplo-SCT								
Perugia; [2]	17	End-stage chemoresistant leukemia	Extensively TCD	TBI + ATG + Cy + Thio	40% NRM; mainly CMV and *Aspergillus* infection	NR	NR	
Perugia; [11]	43	Acute leukemia	Extensively TCD	TBI + ATG + Flu + Thio	The infection-related mortality rate 25–35%	NR	CD4+ >0.1 × 10^9^/L at day 60 and >0.3 × 10^9^/L at day 180	
Lilleri et al.; [12]	48	Malignant or nonmalignant hematological diseases	T cell-depleted peripheral blood CD34+ progenitor cells	ATG + TBI or chemotherapy	9% in R+ or 8% in R− (1-year)	4% in R− and 83% in R+	61% recipients reconstituting CMV-CTL within the first 3 months	Young patients
Chen et al.; [13]	22	Refractory hematological malignancies	Mobilized peripheral blood stem cells depleted of CD3+ cells	Flu + Thio + Mel + OKT3	NR	1/22 patients developed CMV infection	The median number of CD4+ and CD8+ T cells was about 0.2 × 10^9^/L and above 0.1 × 10^9^/L at 3 months	Pediatric recipients
Federmann et al.; [14]	28	Hematological malignancies	CD3/CD19-depleted grafts	Flu or (Clo) + Thio + Mel + OKT-3	NR	Eight of 28 patients had cytomegalovirus reactivation	A median of 205 CD3+ cells/*μ*L, 70 CD3+ CD4+ cells/*μ*L, and 66 CD3+ CD8+ cells/*μ*L on day 100	
Pérez-Martínez et al.; [16]	30	Acute leukemia	CD3/CD19-depleted	Flu + Bu + Thio + mP	23% NRM (7/30)	Two of 30 patients have died because of CMV pneumonia	A median of 167 ± 64/*μ*L CD4+ cells versus 364 ± 174/*μ*L CD8+ cells on day 30, 155 ± 47/*μ*L versus 410 ± 119/*μ*L on day 60, and 217 ± 72/*μ*L versus 537 ± 192/*μ*L on day 90.	Children
Unmanipulated haplo-SCT								
Peking University; [21]	50	Hematological diseases	G-CSF-primed bone marrow and unmanipulated PBSCs	Ara-C + Bu + Cy + simustine + ATG	19.5 ± 6.0% NRM (2-year)	The cumulative incidence of CMV antigenemia in the early posttransplant phase was 49.9 ± 7.2%	CD4+ T cells at 152.91 (13.29–579.63)/*μ*L on day 90, 163.28 (23.29–875.60)/*μ*L on day 180, and 277.49 (16.91–579.48)/*μ*L on day 365; CD8+ T cells at 672.79 (48.23–2,556.01) on day 90, 918.42 (115.00–4,047.91)/*μ*L on day 180, and 884.16 (175.84–2,441.58)/*μ*L on day 365	
Peking University; [22]	42	Malignant hematological disorders	G-CSF-primed bone marrow and unmanipulated PBSCs	Ara-C + Bu + Cy + simustine + ATG	24% NRM (10/42)	The cumulative incidence of CMV reactivation was 87.67% (75.70–95.48%); 5 of them had CMV disease (day 22–50).	The CD8+ T cell count equaled that of controls at day 60, and the median number of CMV-CTL cells was comparable to that of controls from day 30 to day 360	
Kurokawa et al.; [24]	66	Hematologic malignancies	Unmanipulated PBSCs and/or bone marrow	ATG + BU + Mel with TBI or Flu	11% NRM (7/66)	CMV antigenemia occurred in 45 of 57 evaluable patients	CD3+ >1600/*μ*L at day 180 and CD8+ >1200/*μ*L at day 180. The lowest numbers of CD3+, CD4+, and CD8+ T cells were seen at 1 month after transplantation but all continued to rise until 6 months after transplantation.	
Lee et al.; [25]	83	Acute leukemia and myelodysplastic syndrome	Unmanipulated PBSCs	BU + Flu + ATG	18% (95% CI, 12%–29%) TRM	Fifty-eight of 72 evaluated patients (81%) had CMV pp65 antigenemia.	CD8+ lymphocyte counts exceeded pretransplantation levels at 2 months, and >90% of patients maintained counts >200/*μ*L at 3 months after transplantation. 12 months after transplantation, 24 of 27 patients (89%) had CD4+ lymphocyte counts more than 200/*μ*L.	
Kanda et al.; [26]	12	Hematologic malignancies	Unmanipulated PBSCs	Alemtuzumab + TBI or Flu based	17% NRM (2/12)	Ten of the 12 patients experienced CMV reactivation, and CMV disease was observed in three patients	CD3+/CD4+ and CD3+/CD8+ T cells were strongly suppressed within 2 months after haploidentical peripheral blood SCT but recovered on day 90. CMV-CTLs were detected on day 90 at 0.03–0.25% of CD8+ T cells	
Rizzieri et al.; [27]	49	Hematologic malignancies or marrow failure	Unmanipulated PBSCs	Flu + Cy + Alemtuzumab	31% NRM (15/49)	Twenty-five percent of patients experienced a severe infection, whereas 86% experienced reactivated CMV	The median number of CD4+ and CD8+ T cells was about 100/*μ*L and 400/*μ*L at 3 months for transplant recipients without GVHD.	

TCD: T cell-depleted; PBSC: peripheral blood stem cell; NRM: nonrelapse mortality; TRM: treatment-related mortality; CMV: cytomegalovirus; R−: CMV-negative recipients; R+: CMV-positive recipients; CMV-CTL: cytomegalovirus-specific T cells; NR: not reported; TBI: total body irradiation; ATG: antithymocyte globulin; Cy: cyclophosphamide; Thio: thiotepa; Flu: fludarabine; Mel: melphalan; Clo: clofarabine; Bu: busulfan; mP: methylprednisolone; Ara-C: cytosine arabinoside.
